# Self-Organization of Genome Expression from Embryo to Terminal Cell Fate: Single-Cell Statistical Mechanics of Biological Regulation

**DOI:** 10.3390/e20010013

**Published:** 2017-12-28

**Authors:** Alessandro Giuliani, Masa Tsuchiya, Kenichi Yoshikawa

**Affiliations:** 1 Environment and Health Department, Istituto Superiore di Sanitá, 00161 Rome, Italy; 2 SEIKO Life Science Laboratory, SRI, Osaka 540-659, Japan; 3 Systems Biology Program, School of Media and Governance, Keio University, Fujisawa 252-0882, Japan; 4 Faculty of Life and Medical Sciences, Doshisha University, Kyotanabe 610-0394, Japan

**Keywords:** early embryo development, reprogramming, single-cell differentiation, single-cell genome dynamics, self-organization, autonomous self-organized criticality, genome avalanche, statistical thermodynamics, critical transition state

## Abstract

A statistical mechanical mean-field approach to the temporal development of biological regulation provides a phenomenological, but basic description of the dynamical behavior of genome expression in terms of autonomous self-organization with a critical transition (Self-Organized Criticality: SOC). This approach reveals the basis of self-regulation/organization of genome expression, where the extreme complexity of living matter precludes any strict mechanistic approach. The self-organization in SOC involves two critical behaviors: scaling-divergent behavior (genome avalanche) and sandpile-type critical behavior. Genome avalanche patterns—competition between order (scaling) and disorder (divergence) reflect the opposite sequence of events characterizing the self-organization process in embryo development and helper T17 terminal cell differentiation, respectively. On the other hand, the temporal development of sandpile-type criticality (the degree of SOC control) in mouse embryo suggests the existence of an SOC control landscape with a critical transition state (i.e., the erasure of zygote-state criticality). This indicates that a phase transition of the mouse genome before and after reprogramming (immediately after the late 2-cell state) occurs through a dynamical change in a control parameter. This result provides a quantitative open-thermodynamic appreciation of the still largely qualitative notion of the epigenetic landscape. Our results suggest: (i) the existence of coherent waves of condensation/de-condensation in chromatin, which are transmitted across regions of different gene-expression levels along the genome; and (ii) essentially the same critical dynamics we observed for cell-differentiation processes exist in overall RNA expression during embryo development, which is particularly relevant because it gives further proof of SOC control of overall expression as a universal feature.

## 1. Introduction

A fundamental issue in bioscience is to unveil the mechanism that underlies the dynamic control of genome-wide expression through the complex spatial-temporal self-organization of the whole-genome expression to regulate cell-fate determination.

Our recent studies on the dynamics of genome-scale expression in the cell-fate change have demonstrated the potential of a statistical thermodynamic analysis [1,2,3,4]. The thermodynamics’ phenomenological character is at the core of Albert Einstein’s famous statement “A theory is the more impressive the greater the simplicity of its premises is, the more different kinds of things it relates, and the more extended is its area of applicability. Therefore, the deep impression which classical thermodynamics made upon me. It is the only physical theory of universal content which I am convinced will never be overthrown, within the framework of applicability of its basic concepts” [5].

An open-thermodynamic approach clearly reveals how and when the change in a genome-expression state occurs as a thermodynamic event in the disappearance of critical phenomena that arise from an initial state. Furthermore, a detailed global perturbation mechanism for the self-organization of overall expression has been elucidated for both cell differentiation and embryo development [3,4]. This application of statistical thermodynamics concepts in genome expression dynamics [6] is particularly crucial in biology, where the extreme complexity of the system rules out any strict mechanistic approach that considers all of the microscopic regulations involved.

**Figure 1:**
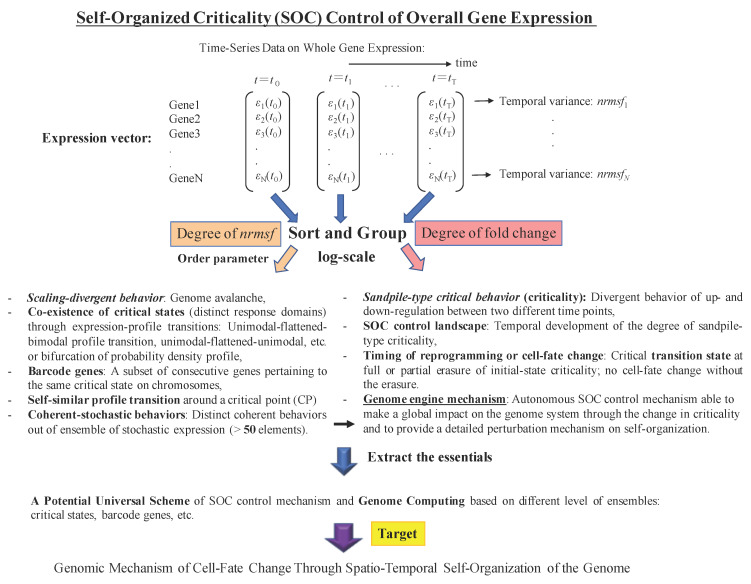
Glossary and strategy of analysis: An extremely schematic view of the data analysis strategy (top) used to reveal SOC gene-expression regulation together with a glossary of the principal features of self-organization behavior highlighted by previous analyses in several biological processes (bottom); scaling divergent behavior (e.g., Figure 1A in [1]; Figure 5F in [2]; Figure 4 in [3]; Figure 1A in [4]); co-existence of critical states (Figures 4, 5 and 8 in [1]; Figures 1 and 2C in [2]; Figure 9 in [3]; Figure 3A in [4]); barcode genes (Figures 8 and 9 in [2]); self-similar profile transitions (Figure 3 in [2]; Figure 6 in [3]); coherent-stochastic behaviors (Figures 7 and 9 in [1]; Figures 5A and 6 in [2]; Figures 10 and 11 in [3]; Figure 3 in [4]); sandpile-type criticality (Figure 2D in [2]; Figures 3A and 4 in [3]); SOC control landscape (Figure 8 in [3]; Figure 2 in [4]); timing of reprogramming (Figures 5, 7B–D and S1 in [3]; Figure 2 in [4]); genome engine mechanisms (Figures 12 and 13 and the Discussion in [3]; Figures 5–7 in [4]).

**Figure 2:**
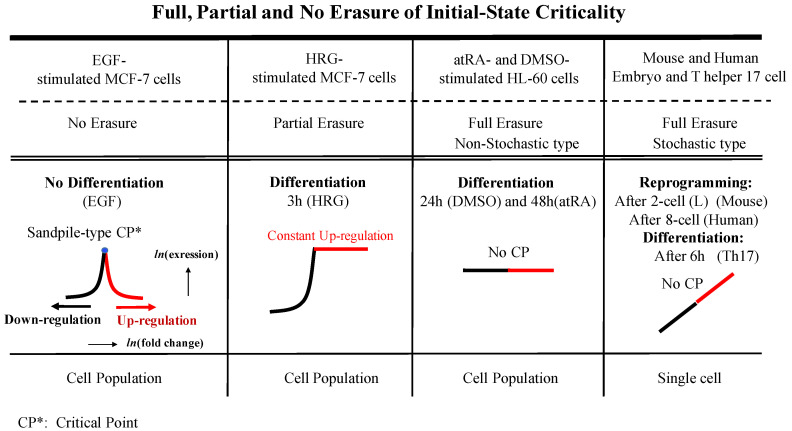
Full, partial and no erasure of initial-state criticality and the timing of reprogramming and cell differentiation (see the details in [3,4]). The figure shows different types of erasure of initial state criticality in embryo development and terminal cell differentiation.

The active process that controls global gene expression in the case of cell-fate determination involves a particular kind of self-organization, called Self-Organized Criticality (SOC) [2,7,8,9], which drives the genome to pass over a critical transition via the mutual interaction among distinct gene-expression states (critical states; see more in [3,4]). This allows for a phenomenological (and thus independent of any mechanistic hypothesis) description of the expression dynamics at the global level. The peculiar self-organization behavior based on SOC has two distinctive features: sandpile-type criticality and scaling-divergent behavior (Figure 1):

(i) In the case of sandpile-type criticality, we observe a critical point (summit of the sandpile) where up- and down-regulation neatly diverge: such behavior emerges spontaneously by the grouping of different gene expressions according to their fold-change. Reprogramming or cell-fate change is observed in terms of both erasure of criticality of an initial organized state such as an early embryo state and the achievement of a new organized configuration (terminal differentiation; see Figure 5: terminal cell differentiation for cell population [3]; Figure 2 in [4] for mouse embryo development; see more in Figure 2).

(ii) Scaling-divergent behavior emerges when genes are grouped according to their normalized expression variability (*nrmsf*: normalized root mean square fluctuation; Appendix A), and thus, with no relation to their specific functional role. This grouping is sufficient to generate both linear (scaling) and divergent domains in the “fold-change” vs. “*nrmsf*” log-log space; the divergent behavior anticipates a transition [10]. The separation of scaling domains (Figure 1 in [4]) induced by a global order parameter, such as *nrmsf*, shows how self-organization occurs in overall expression, and is not confined to a few “master genes”.

To clarify the overall role of SOC in genome expression, as well as our strategy of analysis, Figure 1 presents a general picture of the procedure we adopted together with a short glossary.

The erasure of initial-state criticality can be appreciated by the temporal development of sandpile-type criticality between an initial (fixed) and different states. Figure 2 (below) shows different types of erasure of initial-state criticality, such as for embryo development and terminal cell differentiation. In human and mouse embryo development, the erasure of initial-state criticality exhibits random (stochastic) patterns, i.e., full erasure, while we observe full, partial and no erasure patterns in cell differentiation. The timing of such erasures of initial state criticality is consistent with experimental findings regarding cell differentiation and reprogramming (refer to the Discussion in [3,11] for mouse embryo). In mouse embryo development, erasure of the initial state corresponds to a critical transition state, where the collective behavior of low-variance stochastic gene expression generates autonomous SOC control to allow the reprogramming through a transition state [4]. Furthermore, there exists an SOC control mechanism (genome engine mechanism [3,4]) in which a change in criticality can affect the entire dynamical genome system, and thus the erasure of initial-state criticality suggests a pruning procedure for regulating global gene expression in the initial state.

The erasure of zygote-state criticality in mouse embryo development corresponds to a crucial step in development: elimination of the initial “maternal epigenetic imprinting” of gene regulation. This maternal imprinting corresponds to circulating messenger and regulatory RNA from the egg cell and drives gene regulation in the zygote before the new paternal-plus-maternal genetic information comes into play, to start a new global control that is driven by the new genetic make-up. In the zygote, the new diploid genome is still inactive in a chromatin fully-condensed state, and the metabolism is orchestrated by maternal RNA molecules. Thus, for further development, it is important to erase the previous control to allow for a new start based on the new maternal/paternal mixed genome (see Section 3).

In some sense, this “erasure” path can be considered to be a transformation that proceeds in a direction opposite that for acquisition of a terminal cell fate. Our interpretation of reprogramming in terms of the erasure of mouse epigenetic maternal imprinting at the 2-cell state is consistent with the experimental results [11].

In this report, we investigate whether the same critical dynamics (SOC control of overall expression) as we observed for cell differentiation processes [2,3] exist in overall RNA expression of embryo development and show a characteristic phase transition of the mouse genome under SOC control; the essence of the transition is interpreted with a control parameter of SOC. Our results are particularly relevant to give further proof that SOC control is a universal feature of cell-fate determination and development.

## 2. Results

**Figure 3:**
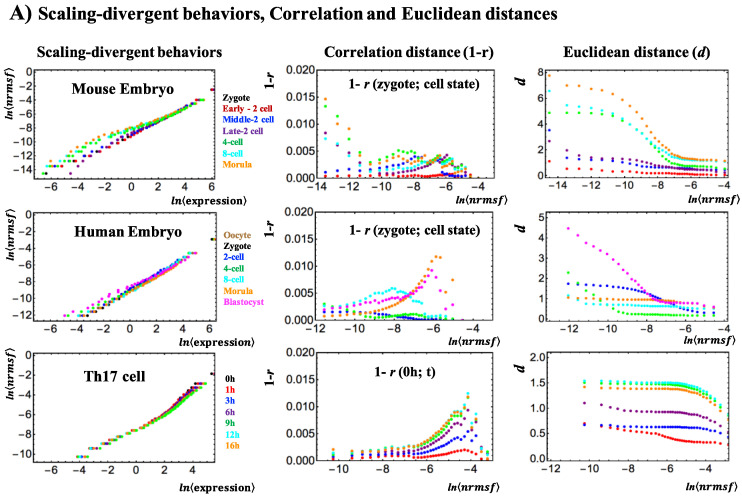

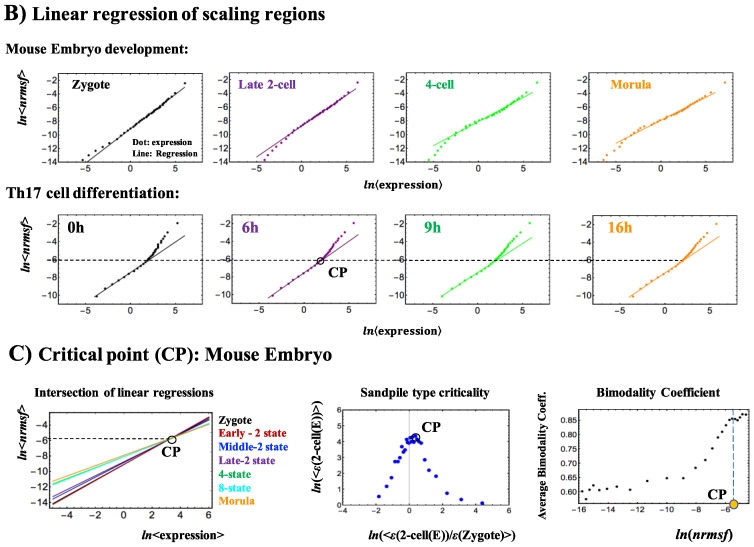
Scaling-divergent behaviors and critical points: (**A**) First column: Genome avalanches, scaling-divergent behaviors in overall expression, as important features of the SOC control of overall expression are evident in the log-log plot of average behaviors: mouse (first row), human (second) embryo development and Th17 immune cell differentiation (third). Second column: Correlation distance, expressed as (1 − *r*), where *r* is the Pearson correlation coefficient between the gene-expression profiles in the zygote and other development states (mouse: first row and human: second row) and between *t* = 0 and other time points (*t*_j_) (Th17). This distance corresponds to the relative change in the expression profile on the whole-genome scale. The results show that there is a difference in scaling-divergent behaviors: in mouse and human embryo, correlation behaviors significantly change at low and middle *nrmsf*, respectively, whereas in terminal Th17 cell, significant change occurs at high *nrmsf*. Third column: Euclidean distance from the initial-state response (zygote state for embryo development and response at *t* = 0 for Th17 cell) shows that two distinct biological processes (reprogramming in early embryo development versus immune cell differentiation) show opposite scaling-divergent behaviors. Scaling behavior (i.e., constant behavior in Euclidean distance) occurs in the ensemble of high-variance RNA expression (region of high *nrmsf*) in early embryo development, and divergent behavior occurs in the ensemble of low-variance RNA expression (region of low *nrmsf*: sub-critical state), whereas the T cell terminal cell fate (single cell) has opposite behaviors. Log-log plots represent the natural logarithm of the group average (< >) of expression (*x*-axis) and *nrmsf* (*y*-axis) (*n* = 485 (mouse), 475 (human), and 375 (Th17) for each dot), where overall expression is sorted and grouped (35 groups) according to the degree of *nrmsf* (Appendix A); (**B**) Linear regression of scaling regions in the scaling-divergent behaviors; mouse embryo development (upper row) and Th17 cell differentiation (lower row); (**C**) critical point: in mouse embryo development, a summit (CP) of sandpile criticality (middle panel) corresponds to a tipping point of transitional behavior of the bimodality coefficient (right) and, furthermore, to the intersection of linear regressions (left) (see more in [4]). This suggests that the CP is fixed during early mouse embryo development. In Th17 cell differentiation, the CP corresponds to the onset of divergent behaviors, which is also fixed in single-cell differentiation (see (**B**)).

SOC control of overall expression [3] represents the self-organization of coexisting distinct response expression domains through a critical transition. Temporal variance expression (normalized root mean square fluctuation: *nrmsf*; see Appendix A) acts as an order parameter in self-organization. Figure 3A shows the “angular” (order only) and “Euclidean” (global) scaling in the scaling-divergent behaviors (Figure 3A: left panel). The correlation distance is an angular metric (Pearson *r* is the cosine of two vectors, each of which corresponds to a set of expression values), thus encompassing only the “order” information, whereas the Euclidean metrics represent both “size” and “order” information, and there is no possibility of disentangling the two contributions. This result suggests the existence of coherent waves of condensation/de-condensation of chromatin in cell development (*nrmsf* is a proxy for chromatin flexibility), which are transmitted from a region of low expression variability (low *nrmsf* region) in reprogramming of the embryo genome to a region of high expression variability (high *nrmsf*) in terminal cell differentiation.

**Figure 4:**
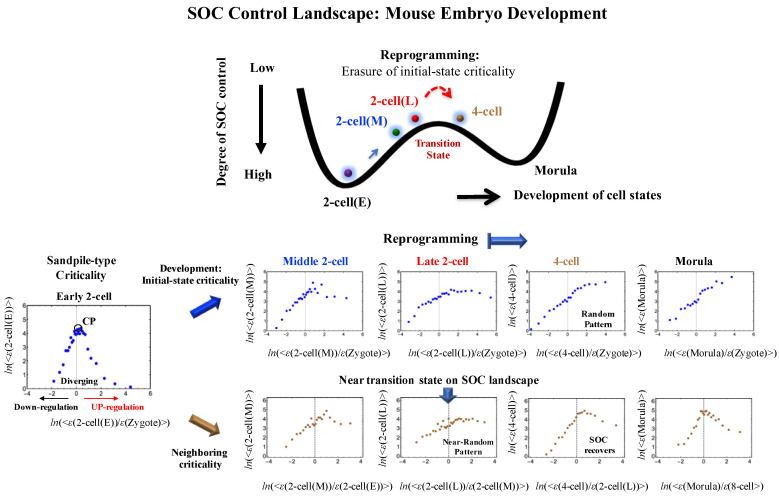
Timing of the genome-state change on the SOC control landscape in a single cell: A change in critical dynamics through sandpile-type criticality (diverging up- and down-regulation at the CP around *ln*(<*nrmsf*>) ~ −5.5), which affects the entire genome-expression dynamics (see details in [4]), appears in the change in overall expression (e.g., fold-change) between different time points. Thus, the erasure of sandpile-type criticality in the zygote state points to the timing of a genome-state change in mouse embryo development. This erasure of zygote criticality (upper row: development of initial-state criticality) occurs after the late 2-cell state to reveal a stochastic expression pattern as a linear correlative behavior (refer to [3]). The near-transition point (see a schematic picture of the SOC landscape: top panel) occurs at the middle-late 2-cell states (lower row: development of neighboring criticality), at which sandpile-type criticality disappears and thereafter recovers. Plots reveal the existence of an SOC control landscape and a transition state at around the middle-later 2-cell states. The *x*- and *y*-axes represent the fold-change in expression and the group average expression. A detailed mechanism of how reprogramming occurs via the interaction of distinct coherent expression states is given in [4], where the collective behavior of stochastic low-variance RNA expression as a generator of autonomous SOC control guides the reprogramming of mouse embryo development.

In the critical dynamics of genome expression, we demonstrated how a change in sandpile-type criticality (Critical Point (CP): the divergence of up- and down-regulation [3]) affects the entire-genome expression system [3,4]. Thus, it is important to find the location of the CP (in terms of mean-field) and to observe how it changes over time. In Figure 3B,C, linear regressions of the scaling region (Figure 3A: left panel) suggest that there is a fixed CP in both mouse early embryo development and single Th17 cell differentiation [4]. In mouse embryo development, the CP (Figure 3C) exists at the boundary of the high-*nrmsf* region (Figure 3A in [4]), whereas in differentiation, the CP—the onset of divergent behavior at scaling-divergent behavior—is at the boundary of the low-*nrmsf* and high-*nrmsf* regions (Figures 4B and 9 in [3]: low for MCF-7 cells and atRA-stimulated HL-60 cells; high for DMSO-stimulated HL-60 cells).

The temporal development of sandpile-type criticality for the initial and neighboring states (Figure 4) reveals that reprogramming in the mouse embryo follows a clear signature of an approaching critical transition state, where a near-transition state exists at the middle-late 2-cell states. This provides the SOC control landscape, a quantitative (open-) thermodynamic appreciation of the still largely qualitative notion of the epigenetic landscape in reprogramming (see the detailed perturbation mechanism in self-organization regrading reprogramming [4]).

**Figure 5:**
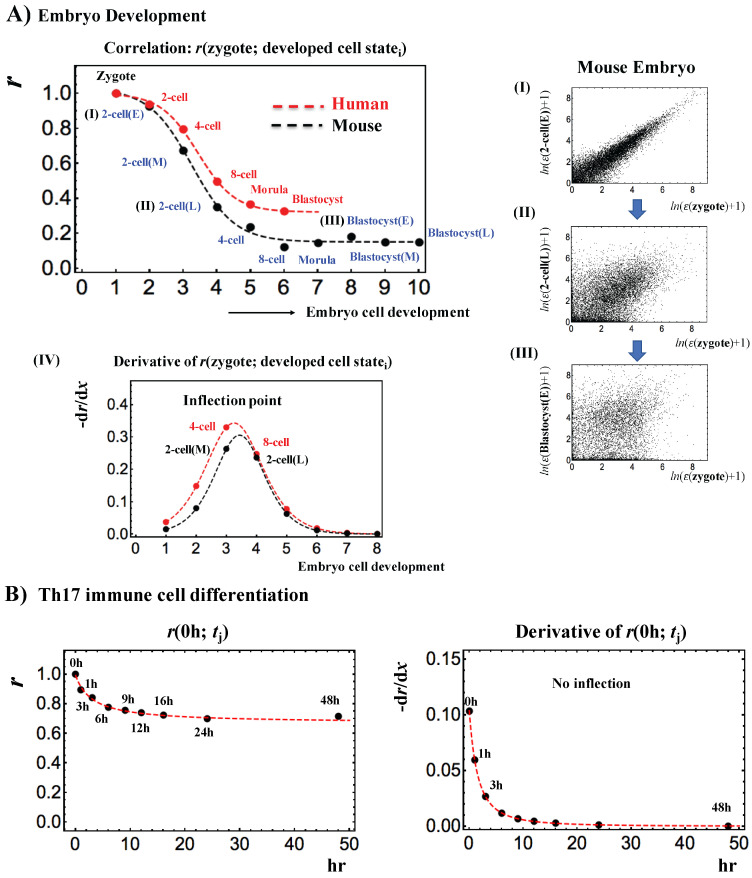
Critical transition revealed through changes in the Pearson correlation between the zygote and cell developed states: (**A**) Pearson correlation for the developed cell state with the zygote exhibits a critical transition as a tangent hyperbolic function, 0.59−0.44 tanh(0.78 x−2.5), (*p* < 10^−3^) (black dash: mouse embryo; red: human embryo) with between-whole expression profiles (right panel): (I) zygote vs. early 2-cell state; (II) zygote vs. late 2-cell state; (III) zygote vs. early blastocyst; (IV: the plot shows that a phase transition occurs at the inflection point (zero second derivative of the tangent hyperbolic function); there is a phase difference between the 4-cell and 8-cell states for human and between the middle and late 2-cell states for mouse. Since there are zero values in RNA expression (Reads Per Kilobase Mapped (RPKM) values), before taking the natural log of expression, we add a value of 1 to all RPKM values for between-whole expression profiles. ε (cell state) represents whole RNA expression at a specific cell state. (**B**) Temporal development of the Pearson correlation of whole expression at *t* = 0 h. It follows 0.67 + 1/(3.10 + *x*) (*p* < 10^−8^; red dashed line) without any inflection (i.e., no phase difference as in embryo development). The (negative) derivatives of Pearson correlation for (**A**,**B**) are taken from the fitting functions.

Next, the temporal development of the Pearson correlation of zygote state expression with that of the developed cell state (Figure 5A) shows a transition, and follows a tangent hyperbolic function with an inflection point. Regarding “phase”, an inflection point represents a phase difference [12,13] between the middle and late 2-cell states in mouse embryo and between 4-cell and 8-cell states in human embryo. Notably, the results of moue embryo support the notion that the most significant perturbation (“energy-flow”) in self-organization occurs at the middle-late 2-cell state (see Figure 7 in [4]) for the new embryonal genome (stochastic pattern: Figure 4: development of initial-state criticality) before passing over a critical transition state (Figure 4: development of neighboring criticality). These results demonstrate that a phase transition occurs in the genome before and after reprogramming through the critical transition state, consistent with a control parameter of the phase transition in SOC control.

In contrast, Figure 5B shows that, there is no evident inflection point in Th17 immune T cell differentiation; the whole expression profile at *t* = 0 h remains high over time, which is also observed in cancer cell differentiation in cell populations [3]. As shown in Figure 3A (divergent behavior of high temporal-variance genes), this suggests that cell differentiation involves more localized gene expression than early embryo development, which is consistent with the view in current molecular biology, i.e., the path toward a terminal cell fate is a path toward increasing order with respect to the undifferentiated state [14].

Finally, these findings are still phenomenological and further studies will be needed to confirm the corresponding material bases of biological regulation. Nevertheless, our results suggest that a statistical approach to biological regulation can provide useful insight into the underlying mechanism of self-regulation/organization of life in cases where the extreme complexity of the system rules out any strict mechanistic approach.

## 3. Discussion

Throughout our studies [1,2,3,4], we have demonstrated that the SOC control of genome expression based on a mean-field approach is rather universal among several distinct biological processes. Furthermore, an SOC control mechanism reveals the details of how and when the cell-fate change, including reprogramming, occurs for several biological processes at both the cell population and single cell levels [3,4]. Here, we focus on the phase transition, the biological mechanisms of sandpile-type criticality and backward reprogramming (iPS cells).

### 3.1. The “Phase Transition”

Our results are mainly phenomenological and do not include detailed arguments such as a quantitative evaluation of the partition function through a path-integral. Our findings regarding a “phase transition” in genome expression are based on a so-called symmetry argument (i.e., Landau’s symmetry argument [15] and symmetry breaking [16,17]), which has become an indispensable strategy and framework in statistical physics. With regard to genome expression, we found that temporal-variance expression (*nrmsf*; see Appendix A) acts as an order parameter in self-organization [1,2,3,4]. According to the degree of *nrmsf*, overall expression exhibits symmetry-breaking in the expression profile, such as the unimodal-flattened unimodal-bimodal transition in MCF-7 cancer cells) [1,2,3] and the unimodal-flattened unimodal-unimodal transition in HL-60 cancer cells [3]. This shows that there are distinct phases (called “critical states”) in the expression profile along the order parameter. Furthermore, the good coincidence of peaks between probability density and frequency distribution suggests the existence of an energy-like potential that encompasses thousands of mRNA transcriptional reactions.

Distinct coherent behaviors, which represent different phases, emerge from stochastic expression (coherent-stochastic behaviors as the collective behaviors of groups with more than around 50 genes (mean-field approach) [18]. The expression profile (bimodality coefficients) of groups according to the order parameter (*nrmsf*) in different biological processes follow distinct transitions such as a tangent hyperbolic function and a step-functional-like transition with tipping points in overall expression (MCF-7 cells: Figure 9A and HL-60 cells: Figure 9B in [3]; mouse embryo: Figure 3C).

These results show that SOC control of overall expression through a critical transition explains the self-organization of genome expression with the coexistence of distinct critical states at a certain time point, which is different from a (first- or second-order) phase transition in an equilibrium system, i.e., a phase transition in the overall expression from one critical state to another through a critical transition.

Regarding mouse embryo cell development, programming occurs by overcoming a transition state on the SOC control landscape (Figure 4), which is further supported by the existence of phase differences regarding programming (Figure 5A). This suggests the existence of another layer of phase transition of a macro-state (genome state) (i.e., genome state before and after programming) along a control parameter through SOC control of overall expression, where distinct critical states (found by us) are “micro-critical states”.

### 3.2. The Biological Mechanisms of Sandpile-Type Criticality

It is worth discussing the view of biological “mechanisms” implied by sandpile-like criticality (and more general statistical thermodynamics approaches). The term “sandpile” comes from the fact that, when a real sandpile reaches a critical height, the addition of a single grain is sufficient for avalanches to start. While the site of the first avalanche is unpredictable, the value of the critical threshold (and the time to reach it at a given growth rate) can be known with great precision. If we maintain (as in the present paper) a largely phenomenological perspective, we can focus on understanding a general view such as the occurrence of avalanches (with gene expression playing the role of sand grains) and the critical height (critical point of divergence), and discard the quest for “local interaction” (the last sand grain, here a specific gene) that initiates the transition.

It is important to stress that our interpretation of the erasure of epigenetic maternal imprinting at the 2-cell state is consistent with the results of [11] demonstrating that, in mouse, “the oocyte-to-embryo transition’’ is driven by degradation of maternal mRNAs, which results in loss of oocyte identity, and reprogramming of gene expression during the course of zygotic gene activation, which occurs primarily during the 2-cell stage and confers blastomere totipotency” [11]. This points to a massive (the entire genome expression is affected) change that is not confined to specific master genes.

This was the view of embryologists in the first half of the last century (who clearly could refer only to observed phenotypic changes and not to gene expression) who demonstrated that development was induced by the implantation of an “organizer” (a tiny piece of another embryo), which initiated the “development catastrophe” [19].

In their very interesting works [20,21], the authors try to reconcile the global phenomenological view (explicitly referring to Spemann and Mangold [19]) with a molecular viewpoint of development. The focus is on the ability of *Xenopus* [20] to scale the differentiation patterns with embryo size: the symmetry-breaking of an initial homogeneous cytoplasm into separated differentiation fate compartments relies on the graded distribution of Bone Morphogenetic Protein (BMP) activity across the embryo.

Patterning is driven by a shuttling mechanism, where the BMP ligands are transported by a common BMP inhibitor (Chordin) to the most ventral part of the embryo, thus establishing a sharp, power-law decaying activation profile [20]; the authors are able to develop a mathematical model of the generation of the observed gradient based on a nine-element interaction network. Notably, analogous to our model, there are many possible initial configurations of the network that collapse to the same solution (in this case, a correctly identified gradient); this finding is consistent with attractor dynamics [14,22], which we envisage for both development and terminal differentiation, and opens a bridge that links classical mechanistic and “statistical mechanics-oriented” approaches to biological sciences [6].

Finally, regarding the dynamics of criticality, the critical gene ensemble supporting the CP (summit of sandpile criticality) plays an essential role in reprogramming [3,4], and thus, we will next seek to elucidate how the critical gene ensemble affects the entire genome by focusing on the elucidation of the material basis of the spreading of information across chromatin.

### 3.3. Regarding Backward Reprogramming

In the investigations of the backward reprogramming of iPS cells from somatic cells, a stochastic model [23,24] has been used to explain how reprogramming occurs through the ectopic expression of transcription factors, such as Yamanaka factors, in a probabilistic manner. The stochastic model can be due to temporal-spatial asynchronous molecular reaction events in heterogeneous cell populations [25]. Thus, an investigation of the breakdown of SOC control in the reprogramming of single adult somatic cell and the existence of the corresponding SOC control landscape will be important for understanding how and why a single cell can overcome an epigenetic landscape potential barrier to achieve backward reprogramming (experimental procedure for single iPS cells in [25]). A comparative analysis of the breakdown of SOC control between success and failure of reprogramming in single adult cells could elucidate the underlying molecular mechanism to explain why single somatic cells succeed or fail at achieving reprogramming the genome in a non-probabilistic manner. It is expected that (no) breakdown of SOC control occurs in the (failure) success of single-cell reprogramming, or there may be multiple steps in the breakdown of SOC control. Hence, the findings will be fundamentally different from those obtained by current trial-and-error combinatorial approaches in terms of the combination of enhancers and inhibitors of reprogramming [26]. Moreover, investigation of molecular mechanism for the SOC control of the tumorigenicity of iPS cells (population level) may lead to the identification of key tumor-inducing mechanisms.

These findings are expected to open new avenues for clinical applications in humans and tissue engineering.

## Appendix A.

### Appendix A.1. Biological Datasets

We analyzed mammalian RNA-Seq data:

(i) Early embryonic development in human and mouse developmental stages in RPKM (Reads Per Kilobase Mapped) values; GEO ID: GSE36552 (human: *N* = 20,286 RNAs) and GEO ID: GSE45719 (mouse: *N* = 22,957 RNAs), which have seven and ten embryonic developmental stages (experimental details in [27,28], respectively):

Human: oocyte (*m* = 3), zygote (*m* = 3), 2-cell (*m* = 6), 4-cell (*m* = 12), 8-cell (*m* = 20), morula (*m* = 16) and blastocyst (*m* = 30),
Mouse: zygote (*m* = 4), early 2-cell (*m* = 8), middle 2-cell (*m* = 12), late 2-cell (*m* = 10), 4-cell (*m* = 14), 8-cell (*m* = 28), morula (*m* = 50), early blastocyst (*m* = 43), middle blastocyst (*m* = 60) and late blastocyst (*m* = 30), where *m* is the total number of single cells.

(ii) T helper 17 cell differentiation from mouse naive CD4+ T cells in RPKM values, where Th17 cells are cultured with anti-IL4, anti-IFNγ, IL-6 and TGF-β (details in [29]); GEO ID: GSE40918 (mouse: *N* = 22,281 RNAs), which has nine time points: *t*_0_ = 0, *t*_1_ = 1, 3, 6, 9, 12, 16, 24, *t_T_*_=8_ = 48 h. For each time point, sample numbers are as follows: GSM1004869-SL2653 (*t* = 0 h); GSM1004941-SL1851 (*t* = 1 h); GSM1004943-SL1852 (*t* = 3 h); GSM1005002-SL1853 (*t* = 6 h); GSM1005003-SL1854 (*t* = 9 h); GSM1004934-SL1855 (*t* = 12 h); GSM1004935,6,7-SL1856, SL8353, SL8355 (*t* = 16 h; average of three data); GSM1004942-SL1857 (*t* = 24 h); GSM1004960-SL1858 (*t* = 48 h).

RNAs with RPKM values of zero over all of the cell states were excluded. Random real numbers in the interval [0, *a*] generated from a uniform distribution were added to all expression values in the analysis of sandpile criticality (Figure 4; no addition of random numbers in the rest of the figures). This procedure avoids the divergence of zero values in the logarithm. The robust sandpile-type criticality through the grouping of expression was checked by changing a positive constant: *a* (0 < *a* < 10); we set *a* = 0.01. Note: The addition of large random noise (*a* >> 10) destroys the sandpile CP.

### Appendix A.2. SOC Control Mechanism of the Cell-Fate Change

We previously demonstrated that the critical dynamics of self-organizing whole-genome expression (SOC) play essential roles in determining the cell-fate change in distinct biological processes at both the single-cell and population (tissue) levels [3].

In SOC-controlled self-organization (in terminal cell differentiation), *nrmsf* (normalized root mean square fluctuation) acts as an order parameter for the self-organization of whole gene expression [2,3,4]. We can examine if *nrmsf* acts as an order parameter in early embryo development, which is determined by dividing *rmsf* by the maximum overall {*rmsf_i_*}:

(A1) $${rmsf}_{i}=\sqrt{\frac{1}{S}\sum_{j=1}^{S}{\left(\varepsilon_{i}\left(s_{j}\right)-\langle \varepsilon_{i}\rangle \right)}^{2}},$$

where *rmsf_i_* is the *rmsf* value of the *i-*th RNA expression, which is expressed as *ε_i_*(*s_j_*) at a specific cell state *s_j_* (e.g., in mouse, *S* = 10: *s*_1_ *=* zygote, early 2-cell, middle 2-cell, late 2-cell, 4-cell, 8-cell, morula, early blastocyst, middle blastocyst and *s*_10_ *=* late blastocyst), and $\left\langle \varepsilon_{i}\right\rangle$ is its expression average over the number of cell states. Regarding self-organization with critical dynamics, we investigated averaging behaviors in *nrmsf* and in the fold-change in expression, where we observed a stochastic resonance effect in the terminal cell-fate process. For methodological details, refer to our previous works [2,3].
